# Greatly attenuated experimental autoimmune encephalomyelitis in aquaporin-4 knockout mice

**DOI:** 10.1186/1471-2202-10-94

**Published:** 2009-08-06

**Authors:** Lihua Li, Hua Zhang, AS Verkman

**Affiliations:** 1 Departments of Medicine and Physiology, University of California, San Francisco, California, USA

## Abstract

**Background:**

The involvement of astrocyte water channel aquaporin-4 (AQP4) in autoimmune diseases of the central nervous system has been suggested following the identification of AQP4 autoantibodies in neuromyelitis optica, an inflammatory demyelinating disease.

**Results:**

We investigated the involvement of AQP4 in disease severity in an established mouse model of experimental autoimmune encephalomyelitis (EAE) produced by immunization with myelin oligodendrocyte glycoprotein (MOG_35–55_) peptide. EAE was remarkably attenuated in AQP4 null mice compared to identically treated wildtype mice. Whereas most wildtype mice developed progressive tail and hindlimb paralysis, clinical signs were virtually absent in AQP4 null mice. Brain and spinal cords from AQP1 null mice showed greatly reduced mononuclear cell infiltration compared to wildtype mice, with relatively little myelin loss and axonal degeneration.

**Conclusion:**

The reduced severity of autoimmune encephalomyelitis in AQP4 deficiency suggests AQP4 as a novel determinant in autoimmune inflammatory diseases of the central nervous system and hence a potential drug target.

## Background

Aquaporin-4 (AQP4) is a water-selective channel expressed in plasma membranes of astrocytes throughout the central nervous system (CNS), particularly at astrocyte foot processes at the blood-brain barrier and brain-cerebrospinal fluid interfaces [1,2]. AQP4 facilitates water movement in the brain and spinal cord, astrocyte migration, and neuroexcitatory phenomena (reviewed in ref. [3]). Mice lacking AQP4 manifest remarkable phenotype differences from wildtype mice in models of cytotoxic [4] and vasogenic [5] cerebral edema, brain injury associated with glial scarring [6], epilepsy [7] and cortical spreading depression [8]. Structural data on AQP4 from electron crystallography suggested a possible new role of AQP4 in cell-cell adhesion [9,10], though subsequent experimental studies did not confirm this role [11].

Another potential new role for AQP4 that is unrelated to its cell membrane water transport function was suggested by the discovery of circulating autoantibodies against AQP4 in most patients with the inflammatory demyelinating disease neuromyelitis optica (NMO) [12]. Indirect evidence, including correlations of NMO-IgG titer with disease severity, and clinical benefit of plasmapheresis and immunosuppression, has suggested that NMO-IgG causes NMO (reviewed in refs. [13-15]). How circulating NMO-IgG and CNS AQP4 expression promote inflammation and cause demyelinating lesions in the central nervous system is the subject of intense speculation. Recently, increased AQP4 expression was found in brain and spinal cord in experimental autoimmune encephalomyelitis (EAE), providing further support for the possible involvement of AQP4 in CNS inflammation [16].

Motivated by the potential involvement of AQP4 in an inflammatory demyelinating CNS disease, we investigated the role of AQP4 in EAE using a well-established mouse model of EAE produced by immunization with a peptide against myelin oligodendrocyte glycoprotein (MOG) [17]. Studies were done comparing clinical outcome and CNS histology in wildtype vs. AQP4 knockout mice, which have normal brain microanatomy, blood-brain barrier integrity, and baseline intracranial pressure [4,18,19]. We found remarkably less severe EAE in mice lacking AQP4, providing evidence for a novel role of AQP4 in neuroinflammation.

## Results

**Figure 1:**
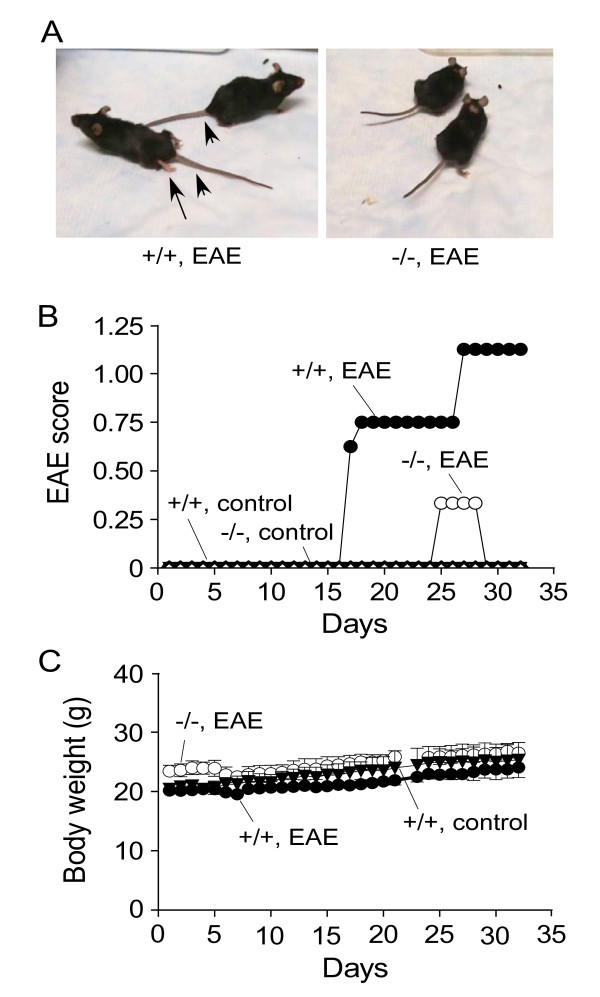
**Clinical assessment of mice following EAE induction by MOG peptide**. **A**. Photographs of EAE-induced wildtype (+/+) and AQP4 null (-/-) mice at 19 days after initial MOG immunization. Arrowhead, tail paralysis; arrows, hindlimb weakness/paralysis. See Supplemental Materials for movie. **B**. EAE clinical score (see Methods) for EAE-induced and control wildtype and AQP4 null mice. **C**. Mouse body weight. Differences in body weight gain not significant.

EAE was induced in wildtype and AQP4 null mice in a C57/bl6 genetic background by immunization with MOG_35–55 _peptide. Most wildtype mice developed progressive tail and hindlimb weakness, generally seen by 16 days after the initial immunization, and progressing in some mice to complete hindlimb paralysis. In contrast, the AQP4 null mice did not develop clinical signs except for transient tail weakness in one MOG-treated AQP4 null mouse. Control wildtype and AQP4 null mice, which were identically treated except for exclusion of MOG peptide, did not develop clinical signs. Fig. 1A shows photographs of two MOG-treated wildtype and two AQP4 null mice, demonstrating tail and hindlimb weakness in the wildtype mice. A movie showing the difference is provided [see Additional file 1]. Fig. 1B summarizes the clinical scores, which were assessed without knowledge of genotype information, showing remarkably attenuated EAE clinical signs in the AQP4 null mice. Fig. 1C shows similar body weights of MOG-treated wildtype and AQP4 null mice, and control mice.

To verify that wildtype and AQP4 null mice responded appropriately to the MOG immunizations, the right hindpaws of a subset of mice were injected with 50 μg MOG_35–55 _at 15 days after the initial immunization, according to standard procedure [20,21]. Marked and comparable swelling was seen in the MOG-treated wildtype and AQP4 null mice, with no swelling seen in control mice (data not shown).

**Figure 2:**
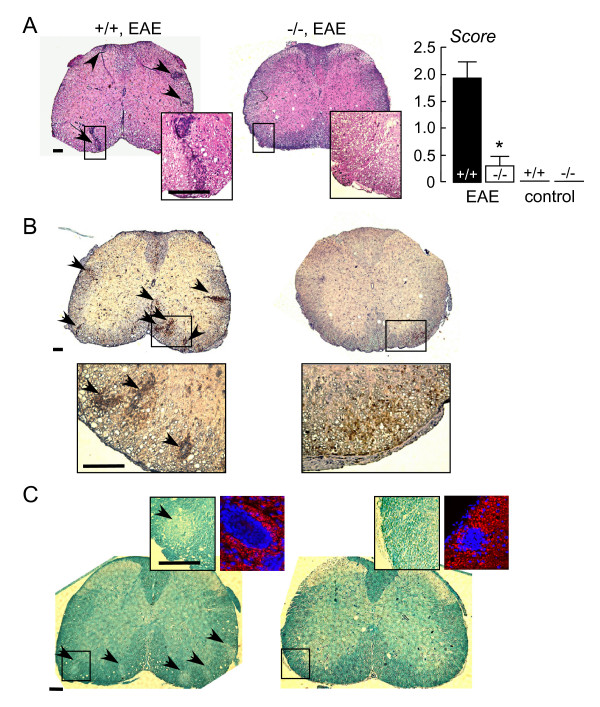
**Spinal cord following EAE induction**. **A**. (left) Representative hematoxylin and eosin stained spinal cord sections (at L_1 _level) from EAE-induced wildtype (+/+) and AQP4 null (-/-) mice, shown at low and high (in boxes) magnifications. Arrows denote areas of mononuclear cell infiltration. All scale bars: 200 μm. (right) Histological score (see Methods) (S.E., p < 0.001). **B**. CD45 immunohistochemistry. Arrows denote CD45-positive lymphocytes in infiltrates. **C**. Luxol fast blue staining. Arrows denote regions of myelin loss. Left inset shows high magnification of Luxol fast blue staining. Right inset shows loss of NF200 immunofluorescence (red) within infiltrates (nuclei stained blue with DAPI).

Spinal cord and brains of MOG-treated and control mice were assessed at 32 days after initial MOG immunization. Fig. 2A (left) shows hematoxylin and eosin staining of spinal cord of MOG-treated wildtype and AQP4 null mice. Mononuclear cell infiltrates were consistently seen in MOG-treated wildtype mice, but were infrequent or absent in AQP4 null mice (and absent in all control mice). Fig. 2A (right) summarizes histological scores obtained from assessment of sections from all mice by two investigators who were blinded to genotype and treatment information. A very significant difference was found. Fig. 2B shows immunohistochemistry for the lymphocyte marker CD45. The positive staining of mononuclear cell infiltrates confirms their cellular identity as lymphocytes. Fig. 2C shows staining for myelin by Luxol fast blue. All MOG-treated wildtype mice showed multiple areas of myelin loss in white mater of spinal cord, with remarkably fewer or no areas of myelin loss in MOG-treated AQP4 null mice.

Immunofluorescence with anti-NF200 antibody revealed axonal loss in white mater in lesions containing cell infiltrates where reduced myelin staining was seen (Fig. 2C, right insets).

**Figure 3:**
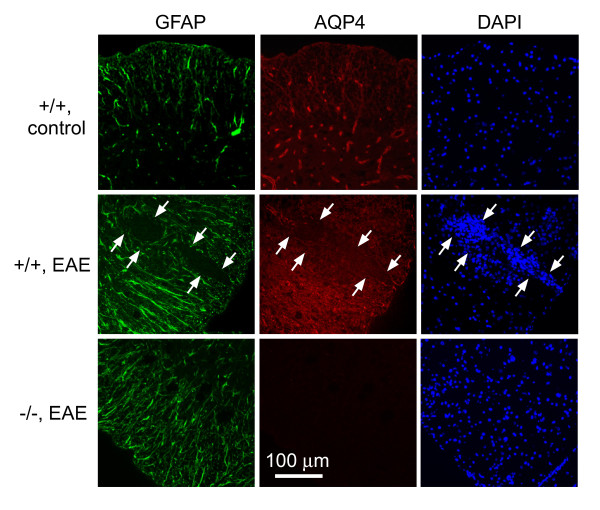
**GFAP and AQP4 in spinal cord**. GFP immunofluorescence (green) and AQP4 immunofluorescence (red) shown with DAPI (blue) stained nuclei. Representative sections shown from EAE-induced wildtype (+/+) and AQP4 null (-/-) mice, and control wildtype mice. Arrows point toward EAE lesion.

GFAP and AQP4 immunofluorescence is shown in Fig. 3. AQP4 expression was seen mainly in GFAP-positive astrocytes in control wildtype mice. EAE induction in wildtype mice produced increased GFAP and AQP4 expression around lesions (demarcated by arrows), but near absence of GFAP and AQP4 immunofluorescence within lesions. AQP4 immunofluorescence was absent in AQP4 null mice, as expected, though there was often seen a mild increase in GFAP immunofluorescence.

**Figure 4:**
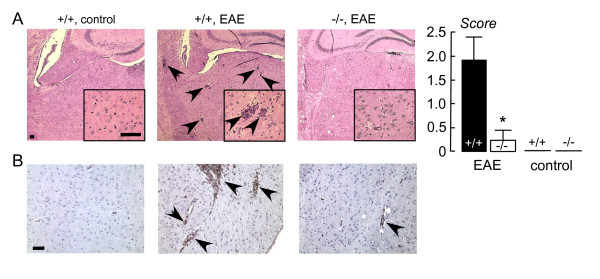
**Brain following EAE induction**. **A**. (left) Representative hematoxylin and eosin stained brain sections from EAE-induced wildtype (+/+) and AQP4 null (-/-) mice, and control (non-MOG-treated) wildtype mice. Arrows denote mononuclear cell infiltrates. All scale bars: 100 μm. (right) Histological score (S.E., p < 0.001). **B**. CD45 immunohistochemistry. Arrows denote CD45-positive lymphocytes.

Fig. 4A shows representative hematoxylin and eosin-stained sections of brain (left) and a summary of histological scores (right). As in spinal cord sections, clearly more mononuclear cell infiltration was seen in the MOG-treated wildtype mice. The cell infiltrates in brain were CD45 positive (Fig. 4B), as seen in spinal cord.

## Discussion

Our data show marked attenuation in the severity of EAE in mice lacking AQP4. The reduced clinical severity, with virtual absence of clinical signs, is consistent with the reduced inflammatory cell infiltration in brain and spinal cord of AQP4 null mice. Whether AQP4 is similarly involved in other neuroinflammatory diseases of the CNS is unknown. We previously reported improved clinical outcome in a mouse model of bacterial meningitis, with greatly reduced meningeal inflammation [22], though it was not possible to isolate effects of AQP4 on inflammation versus brain water accumulation and intracranial pressure elevation. The EAE model here is not associated with significant brain swelling or intracranial pressure elevation.

The mechanisms are not known by which AQP4 expression is associated with increased inflammation in EAE. The association of NMO with AQP4 autoantibodies suggests the possibility of a local inflammatory response involving AQP4. Whether an immune response directly against AQP4 occurs in EAE is not known, nor is it known whether AQP4 expression might exacerbate the astrocyte response to injury because of increased cell swelling and delayed K^+ ^uptake from the extracellular space. Another possibility, based on AQP4 biology, is local alterations in brain water content in the region of the inflammatory lesions. Other possible mechanisms include AQP4-dependent differences in the concentrations of excitatory neurotransmitters, in astrocyte activation and migration, and in inflammatory cell movement into lesions across the blood-brain barrier and brain parenchyma. Investigation of these possibilities will likely present a considerable challenge.

## Conclusion

In conclusion, our studies implicate AQP4 as a novel determinant of the severity of EAE. The increased AQP4 expression reported in EAE around inflammatory lesions likely amplifies the response in a positive feedback manner, increasing the difference in response between wildtype and AQP4 null mice. As such, compounds that interfere with the upregulated expression of AQP4, or perhaps that reduce AQP4 function or plasma membrane expression, may be of clinical benefit in selected neuroinflammatory diseases of the CNS.

## Methods

### Mice

AQP4 null mice in a C57BL/6 genetic background were obtained by >10 back-crosses of mice generated by targeted gene disruption (originally in a CD1 genetic background [18]). Studies were performed on eight-to-twelve week old weight-matched, female C57BL/6 wildtype and AQP4 null mice. Mice were maintained in air-filtered cages and fed normal mouse chow in the U.C.S.F. Animal Care facility. All procedures were approved by the U.C.S.F. Committee on Animal Research.

### Peptide

MOG_35–55 _(single letter amino acid code: H-MEVGWYRSPFSRVVHLYRNGK-OH) was synthesized by Biomatik (Wilmington, DE). The peptide was > 98% pure.

### Induction and clinical scoring of EAE

A 1:1 emulsion was made containing 400 μg MOG_35–55 _in complete Fruend's adjuvant supplemented with 4 mg/ml *Mycobacterium tuberculosis H37Ra *(Sigma). According to standard procedures [20,21], mice were injected 0.1 ml of the emulsion subcutaneously, distributed over three sites along the midline of the back between the shoulders. Mice were injected intravenously with 200 ng pertussis toxin 24 and 48 hours later. Control mice were treated identically except for exclusion of MOG peptide. Mice were boosted subcutaneously with 400 μg MOG_35–55 _in incomplete Fruend's adjuvant one week after the initial immunization. Mice were assess daily for clinical signs using the following scoring: 0, normal mouse; no signs of disease; 1, limp tail or hind limb weakness, but not both; 2, limp tail and hind limb weakness; 3, partial hind limb paralysis; 4, complete hind limb paralysis. Eight MOG-treated wildtype and five AQP4 null mice were used for clinical assessment and histological studies, and four control wildtype and four control AQP4 null mice.

### Histology and immunostaining

At day 32 after initial MOG immunization, mice were anesthetized by intraperitoneal Avertin (125 mg/kg) and perfused transcardially with 4% paraformaldehyde in PBS. Brain and spinal cord were removed and fixed for 24 h in 4% paraformaldehyde. Tissues were dehydrated with increasing concentrations of ethanol, treated with clearing agent, and embedded in paraffin. Sections were deparaffinized and stained using standard procedures with hematoxylin and eosin, and Luxor fast blue (for myelin). Hematoxylin and eosin-stained sections were scored for the severity of inflammation using the following scale: 0, no inflammation; 1, mild inflammation with few mononuclear cells infiltrates; 2, marked inflammation with multiple infiltrates per 100× field; 3, severe inflammation with extensive infiltrates in both white and grey mater.

For CD45 immunocytochemistry, paraffin-embedded sections were cut at 5 μm thickness and deparaffinized, then treated with citrate buffer using microwave antigen retrieval and 3% hydrogen peroxide. Sections were incubated for 2 h with a primary CD45 antibody (1:200, Abcam), then incubated with biotinylated secondary goat anti-rabbit antibody (1:1000, Vector Labs, Burlingame, CA), followed by avidin-biotin peroxidase complex (1:1000). Peroxidase labeling was visualized with diaminobenzidine to yield a brown color. For AQP4, GFAP and NF200 immunofluorescence, sections were incubated with mouse anti-GFAP (Sigma, 1:500) and rabbit anti-AQP4 antibody (Chemicon, Temecula, CA, 1:200), or rabbit anti-NF200 antibody (Chemicon, 1:200), and detected by using fluorescent goat-anti-mouse and goat-anti-rabbit secondary antibodies (1:200, Molecular Probes). Cell nuclei were stained blue with 4',6-diamidino-2-phenylindole (DAPI).

## Abbreviations

AQP4: aquaporin-4; CNS: central nervous system; EAE: experimental autoimmune encephalomyelitis; MOG: myelin oligodendrocyte glycoprotein; NMO: neuromyelitis optica; NF200: neurofilament 200; DAPI: 4',6-diamidino-2-phenylindole.

## Authors' contributions

LL performed did all experimental work, and evaluated clinical scores and immunostaining results.

ASV designed and supervised the study and wrote the manuscript.

HZ assisted in experimental work and independently evaluated clinical histology scores.

All authors read and approved the final manuscript.

## Supplementary Material

Additional file 1 **Video of MOG-treated mice**. Real-time video (total 8 s) of MOG-treated wildtype (left) and AQP4 null (right) mice at 19 days after initial MOG_35–55 _immunization. Click here for file
